# Prevalence, incidence, and trends of childhood overweight/obesity in Sub-Saharan Africa: a systematic scoping review

**DOI:** 10.1186/s13690-020-00491-2

**Published:** 2020-10-29

**Authors:** Frederick Inkum Danquah, Monica Ansu-Mensah, Vitalis Bawontuo, Matilda Yeboah, Desmond Kuupiel

**Affiliations:** 1 Department of Public Health, Faculty of Health and Allied Sciences, Catholic University College of Ghana, Fiapre, Sunyani, Ghana; 2 Research for Sustainable Development Consult, Sunyani, Ghana; 3 Department of Public Health Medicine, School of Nursing and Public Health, University of KwaZulu-Natal, Durban, 4001 South Africa

**Keywords:** Childhood, Obesity, Overweight, Prevalence, Incidence, Trends, Sub-Sahara Africa

## Abstract

**Background:**

The growing burden of non-communicable diseases (NDC), particularly in low-and middle-income countries, poses a significant threat to global health. Obesity and overweight constitute major risk factors of NCDs such as heart diseases, diabetes, and kidney disease, and as a result, contribute significantly to the development of chronic morbidities, reduced quality of life, and increased risk of premature death. This study described evidence on the prevalence, incidence, and trends of childhood overweight and obesity in sub-Sahara Africa (SSA).

**Methods:**

We conducted a systematic scoping review employing the Arksey and O’Malley framework, Levac et al. recommendations, and the Joanna Briggs Institute guidelines. To obtain relevant published articles for this review, we performed a comprehensive keywords search in PubMed, Google Scholar, Web of Science, and CINAHL via EBSCOhost platform for studies published between 2009 and June 2019. Guided by the eligibility criteria, title and abstracts, as well as the full-text articles were independently screened in parallel by two investigators. All relevant data were independently extracted by two investigators using a piloted form designed in Microsoft and thematic analysis conducted.

**Results:**

Of the 81 included studies obtained from 250,148 potentially eligible articles, the majority (25) conducted in South Africa followed by 18 in Nigeria. Six studies were conducted in Ethiopia (6), Tanzania (5), Kenya (4), Cameroon (4), Ghana (3), Uganda (2), Mozambique (2), and Sudan (2). One study each was conducted in Botswana, Gambia, Lesotho, Mauritius, Seychelles, Togo, and Zimbabwe. The remaining three articles were multi-country studies. Most (81.5%) of the included studies were cross-sectional surveys and the majority (79) focused on both male and female participants. The majority (80/81) of the included studies reported on the prevalence of childhood overweight/obesity, 8 on the trends of childhood overweight/obesity, and one presented evidence on the incidence of childhood overweight and obesity in SSA.

**Conclusion:**

This review demonstrates limited studies on childhood overweight/obesity in most SSA countries although the included studies suggest an increasing burden. Considering the consequences of childhood obesity, there is a need for more primary researches to inform policies decision and implementation to halt the rise of childhood obesity/overweight in SSA.

**Supplementary Information:**

The online version contains supplementary material available at 10.1186/s13690-020-00491-2.

## Background

Globally, the burden of non-communicable diseases (NCDs) is rising and has been recognized as a global health issue [1]. NCDs are reported to account for over half of all global health problems [2] and contribute substantially to morbidity and mortality [3]. The Global Burden of Disease 2013 study report indicated that NCDs were responsible for about 70% (38.3million) of the 54.9 million deaths worldwide of which about 80% occurred in low-and middle-income countries (LMICs) [2, 4]. Nutritional disorders such as obesity, overweight, underweight, and stunting in childhood and adolescence are associated with adverse health consequences throughout the life-span [5]. The World Health Organization (WHO) defines obesity and overweight as an abnormal or excessive accumulation of body fat that may impair health [6].

Obesity has been described as one of the greatest health challenges and determinants for many chronic diseases and psychosocial problems and is the fifth leading cause of mortality globally [7]. Obesity and overweight result from a complex interplay of genetic, metabolic, behavioral, cultural, and environmental factors [8, 9]. The epidemic of obesity is mostly driven by the global food system including the increased supply of cheap, palatable, energy-dense foods; improved distribution systems to make food much more accessible and convenient; and more persuasive and pervasive food marketing [10]. The body mass index (BMI) is a simple index of weight-for-height defined as a person’s weight in kilograms divided by the square of his height in meters (kg/m^2^) which is commonly used to classify overweight and obesity [11, 12]. BMI percentiles for age and sex have been developed for diagnosing children older than 2 years of age [13, 14], where children with BMI above the 85th and 95th percentiles are classified as having a higher risk of overweight and obesity respectively [15, 16].

Obesity is one of the major risk factors which predisposes children to the development of non-communicable diseases such as heart disease, diabetes, cancers, and kidney disease, and as a result, contributes significantly to the development of chronic morbidities, increased risk of premature death [17, 18], and reduced quality of life [19]. Muthuri et al. [20] asserted that the health risks associated with obesity and overweight are particularly problematic in children due to the potential for long term health consequences. Obese children are more likely to experience breathing difficulties, increased risk of fractures, and hypertension which are early markers of cardiovascular disease, as well as insulin resistance and psychological problems, in addition to higher chances of premature death and disability in adulthood [6].

Lobstein and Jackson-Leach in 2016 estimated that by 2025 some 268 million children aged 5–17 years globally may be overweight, including 91 million obese based on the assumption that no policy interventions prove effective at changing this trend [21]. Available evidence suggests that the situation in sub-Saharan Africa (SSA) is likely to be worsened by the current nutrition and physical activity transition characterized by increased use of energy-saving devices, availability of cheap high-calorie dense foods, and limited participation in physical activity generally [22, 23]. Moreover, many studies have stressed the negative influence of some sociocultural beliefs in which obesity and overweight are revered and seen as a sign of prestige, good life, and economic value [22, 24–26]. Other studies have also pointed to an obvious link between higher NCD/obesity-related mortalities and increasing socioeconomic development among SSA countries [27]. Despite this, to date, no study has mapped evidence on childhood obesity and identified research gaps to the best of our knowledge. We, therefore, sought to systematically search and examine literature and describe the evidence on the prevalence, incidence, and trends of childhood overweight and obesity in SSA.

## Methods

We conducted a systematic scoping review employing the Arksey and O’Malley framework, Levac et al. recommendations, and the Joanna Briggs Institute guidelines [28–30]. This study forms part of a larger study titled: “Mapping evidence on the burden and distribution of childhood obesity in sub-Saharan Africa”. However, the present study aimed at presenting evidence on the prevalence, incidence, and trends of childhood obesity in SSA. A detailed description of this study methods has been published elsewhere [31]. The Preferred Reporting Items for Systematic reviews and Meta-Analyses extension for Scoping Reviews (PRISMA-ScR) Checklist was followed to report this study (Supplementary file 1).

### Identifying the research question

This scoping review sought to answer the following question: What is the evidence on the prevalence, incidence, and trends of childhood obesity in SSA? To determine the eligibility of the review question for this study, the population, exposure, and outcome (PEO) mnemonic was used (Table 1).

**Table 1:** PEO framework for determining the eligibility of the scoping review question

P-Population	Children (persons aged from 2 to 18 years)
E-Exposure	Overweight (children with BMI ≥ 85th percentile)
	Obesity BMI (≥ 95th percentile or ≥ 35 kg/m^2^)
O-Outcome	Prevalence
	Incidence
	Trends/distribution

### Identifying relevant studies

To obtain relevant published articles for this review, we conducted an exhaustive keywords search in PubMed, Google Scholar, Web of Science databases, and CINAHL via EBSCOhost platform. The database search was conducted between May 2019 and June 2019. In consultation with an experienced Librarian, we developed a comprehensive keywords search strategy to enable identification of all relevant articles as found in the published protocol [31]. Boolean terms, AND/OR were used to separate the keywords. Medical Subject Heading (MeSH) terms were also included in the search. The date was limited from 2009 to 2019, and language and study design limitations were removed during the search. We also searched the reference list of included studies for possible relevant articles. Supplementary file 2 presents the complete search strategy in the electronic databases.

### Eligibility criteria and study selection

This study included articles reporting evidence on obesity/overweight in children aged from 2 to 18 years, articles focused on incidence/prevalence/trends of childhood obesity/overweight, and studies conducted in SSA. Also, only studies published between 2009 to 2019 and in English language were included. Furthermore, this review included only quantitative studies utilising internationally recognized criteria for assessing body composition. However, this study excluded studies reporting evidence on obesity prevalence, incidence and trends among people aged less than 2 years or more than 18 years, risk factors of childhood obesity, studies focusing on knowledge of childhood obesity, articles published in French, and systematic reviews and meta-analyses. Studies conducted in countries not classified among SSA as well as in high-income countries were also excluded.

To reduce selection bias, FID and MAM conducted a comprehensive title search in the databases guided by the eligibility criteria. All potentially eligible studies were imported into Mendeley Desktop that was created for this review and duplicates removed. Again, FID and MAM independently screened the abstracts and full-texts using the inclusion and exclusion criteria. Discrepancies among the reviewers following abstract screening were resolved through discussions to build consensus. However, DK addressed the discrepancies between FID and MAM at the full-text screening stage. Subsequently, the inter-rater agreement (Cohen’s kappa coefficient, κ statistic) between the reviewers was calculated after the full-text screening using Stata version 14. An adapted PRISMA (Preferred Reporting Items for Systematic Reviews and Meta-Analysis) flow diagram was employed to document the study selection process [32].

### Charting data

FID and MY independently extracted data using a tabular piloted form designed in Microsoft excel and DK resolved the discrepancies. Elements of the data extraction form included; author and date, study design, country and setting, sample size, age, gender, and significant outcomes reported (prevalence, incidence, and trend). We also extracted the criteria used for the assessment of body composition.

### Collating and summarizing the results

Following the data extraction, a thematic content analysis was conducted. A narrative approach was used to report the findings from the included studies. The findings from the included studies were summarised and manually coded into the following themes: prevalence, incidence, and trends of childhood obesity.

## Results

**Fig. 1:**
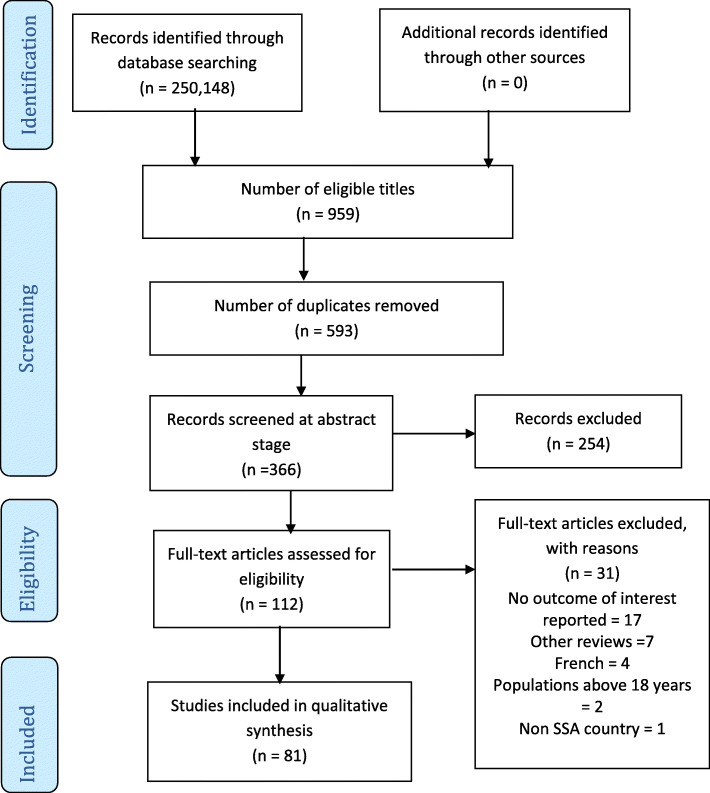
PRISMA chart showing results of literature search and study selection

In all, 250,148 articles were obtained from all databases during the initial search, of which 959 titles met the eligibility criteria at the title screening stage. A total of 593 titles were identified as duplicates and removed using Mendeley Desktop which was used to compile all the eligible titles from the databases. Subsequently, 366 articles underwent abstract screening of which 112 met the inclusion criteria and were included for full-text screening. A total of 31 articles were excluded following the full-text screening stage, and the remaining 81 articles were included for data extraction (Fig. 1). The inter-rater agreement at the full-text screening (Kappa statistic = 0.75, *p* < 0.01) and data extraction (Kappa statistic = 0.87, *p* < 0.01) stages shows there was a moderate to a substantial level of agreement respectively. Of the 31 articles excluded at the full-text screening stage, 17 did not report any of this study’s outcomes of interest [3, 33–48], 7 were review studies [20, 49–54], 4 were published in French though their titles and abstracts were in English [55–58], 2 reported evidence outside this study’s population age range [59, 60], and 1 study was not conducted in of SSA [61].

### Characteristics of the included studies

**Fig. 2:**
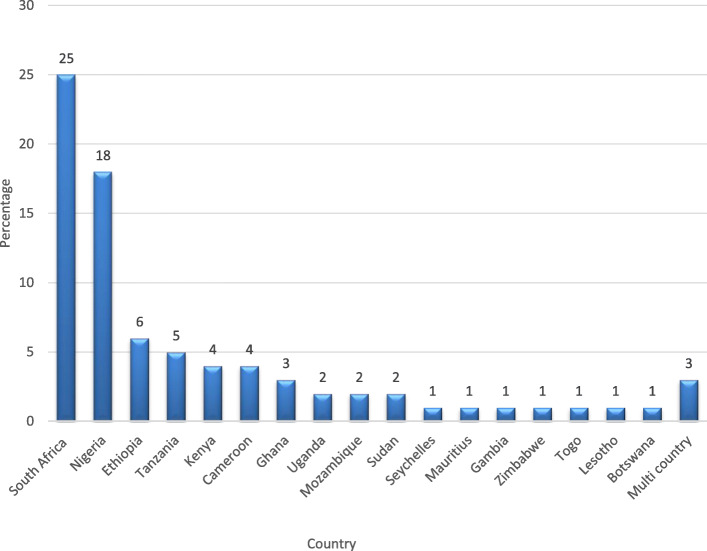
Study settings of the included studies

Out of the 81 included studies, 66 (81.5%) were cross-sectional surveys [16, 24, 62–125]; 8 (9.9%) were secondary data analysis [126–133]; 4 (4.9%) were longitudinal studies [15, 134–136]; one (1.2%) was a national demographic health survey (DHS) [137]; one (1,2%) intervention trial [138]; and one (1.2%) case study [139]. The 81 included studies were conducted in 20 SSA countries of which 17 were single country studies, and 3 multi-country studies. Of the 81 included studies, the majority (25) were conducted in South Africa followed by Nigeria with 18 studies. One of the multi-country studies reported evidence 7 countries of which 4 (Benin, Ghana, Mauritania, and Malawi) were SSA countries [111]. The remaining two multi-country studies reported evidence from two countries each. That is, Kenya and South Africa [110], and Ghana and Uganda [128]. Figure 2 illustrated the distribution of the included studies per country. (Fig. 2).

About 47% (38/81) of the studies were conducted in urban settings; 33.3% (27/81) in both rural and urban areas; 7% (6/81) in rural settings; 3.7% (3/81) in semi-urban settings; 2.5% (2/81) in urban and suburban settings; and 1.2% (1/81) in a peri-urban setting. In about 5% (4/81) of the included studies, there was no indication of the study setting. Most of the included studies 97.5% (79/81) reported on both males and females and 2.5% (2/81) reported evidence on only females. There was no study conducted with only male participants.

Approximately 96% (78/81) of the included studies reported evidence on children older than 5 years whilst about 4% (3/81) reported on children under 5 years. The sample size of the included studies ranged from 56 [139] to 24,391 [63] participants. Of the 81 studies, 39 (48.2%) utilized WHO reference criteria, 26 (32.1%) employed Cole et al. and international obesity task force (IOTF) cut-offs, 6 (7.4%) used BMI cut-of-points, and 3 (3.7%) utilized United States of America Centers for Disease Control and Prevention BMI growth charts. Seven (8.6%) of the 81 included studies compared two or more of the assessment criteria (Table 2).

**Table 2:** Characteristics and findings of the included studies

	Author & date	Study design	Country	Setting	Sample size	Age range (years)	Gender	Outcome reported	Prevalence of overweight	Prevalence of obesity	Criteria for assessment of body composition
1	Armstrong et al., 2011 [63]	Cross sectional	South Africa	Rural, urban	24,391	8–11	Male, female	Prevalence, trend	13.0%	3.3%	IOTF
2	Armstrong et al., 2017 [64]	Cross-sectional	South Africa	Rural, urban	10,285	6–13	Male, female	prevalence	15.4%		IOTF
3	Baumgartner et al., 2013 [138]	Placebo-controlled, double-blind intervention trial.	South Africa	Rural, urban	321	6–11	Male, female	Prevalence	28%		WHO
4	Craig et al., 2013 [65]	Cross-sectional	South Africa	Rural	1519	7–15	Male, female	Prevalence	9.2, 8.1, and 8.0% in males compared to 13.6, 13.4, and 25.8% in females aged 7, 11, and 15 years respectively using WHO 2007 reference criteria 9.2, 8.1, and 8.0% in males compared to 13.6, 13.4, and 25.8% in females aged 7, 11, and 15 years respectively using Cole et al. and IOFT		WHO IOTF NCHS/WHO
5	Feeley et al., 2013 [134]	Longitudinal	South Africa	Urban	1298	13–17	Male, female	Prevalence, trend	8.1% (Males) and 27.0% (Females)		WHO
6	Ginsburg et al., 2013 [135]	Longitudinal	South Africa	Urban	1613	15	Male, female	Prevalence	8.0%(Males) and 25.0% (Females)		IOTF
7	Kimani-Murage et al., 2010 [115]	Cross-sectional	South Africa	Rural	3511	1–20	Male female	Prevalence	18% in females compared to 4% in males		IOTF
8	Kimani-Murage et al., 2011 [66]	Cross-sectional	South Africa	Rural	1848	10–20	Male, female	Prevalence	4% Boys) and 15% (Girls)		IOTF
9	Kruger et al., 2011 [112]	Cross-sectional	South Africa	Rural, urban	2157	1–9	Male, female	Prevalence, trend	10%	4%	WHO
10	Lesiapeto et al., 2016 [126]	Secondary analysis	South Africa	Rural	2485	Under 5	Male, female	Prevalence	16.1%		WHO
11	Lundeen et al., 2015 [127]	Secondary analysis	South Africa	Urban	1172	1–18	Male, female	Prevalence, incidence, trend	Boys = 19.1, 16.4, 9.9, 7.8, 5.7% and Girls = 19.1, 12.2, 14.7, 17.8, and 19.1% at 1–2, 4–8, 11–12, 13–15, and 16–18 years respectively.	Boys = 8.8, 3.0, 6.0, 4.4%, 2.5 and Girls = 8.1, 3.1, 6.4, 7.3, and 7.9% at 1–2, 4–8, 11–12, 13–15, and 16–18 years respectively.	WHO
12	Meko et al., 2015 [116]	Cross-sectional	South Africa	Urban	415	13–15	Male, female	Prevalence	6%		WHO
13	Mokabane et al., 2014 [139]	Case study	South Africa	Peri-urban	56	13–19	Female	Prevalence	12.5%	3.6%	BMI
14	Moselakgomo et al., 2017 [117]	Cross-sectional	South Africa	Rural	1361	9–13	Male, female	Prevalence	Boys = 9.9% (CDC classification) and 2.6% (IOTF criteria) Girls = 10.4% (CDC classification) and 1.0% (IOTF criteria)	Boys = 5.46% (CDC classification) and 0.7% (IOTF criteria) Girls = 5.3% (CDC classification) and 0.6% (IOTF criteria)	CDC IOTF
15	Munthali et al., 2016 [136]	Longitudinal	South Africa	Urban	1824	5–18	Male, female	Prevalence	Girls = late onset overweight (15%) Boys = early onset overweight to normal (6%)	Girls = early onset obesity to overweight (4.8%) Boys = early onset overweight to obese (1.3%)	IOTF
16	Negash et al., 2017 [68]	Cross-sectional	South Africa	Urban	1559	7–18	Male, female	Prevalence	22.9%		IOTF
17	Ngwenya et al., 2017 [67]	Cross-sectional	South Africa	Urban	175	13–19	Male, female	Prevalence	15.4%	8.6%	BMI
18	Pienaar, 2015 [15]	Longitudinal	South Africa	Rural, urban	574	6–9	Male, female	Prevalence, Trend	Did not report this	16.7%	IOTF
19	Pretorius et al., 2019 [69]	Cross-sectional	South Africa	Rural, urban	1785	6–12	Male, female	Prevalence	27.3%		WHO
20	Reddy et al., 2012 [113]	Cross-sectional	South Africa	Rural, urban	4010	Mean = 16.5	Male, female	Prevalence, Trend	Males = rates increased from 6.3% in 2002 to 11.0% in 2008. Females = rates increased from 24.3% in 2002 to 29.0% in 2008	Males = rates doubled 1.6% in 2002 to 3.3% in 2008 Females = rose from 5.0 to 7.5%	IOTF
21	Sedibe et al., 2018 [118]	Cross-sectional	South Africa	Rural, urban	3490	11–15	Male, female	Prevalence	More females overweight and obese at both early and mid-adolescents compared to boys. Early adolescents = (rural: 17.34% vs. 9.52%; urban: 36.15% vs. 27.89%), and mid-adolescents = (rural 22.33% vs. 5.50%; urban: 28.5% vs. 12.82%)		WHO
22	Steyn et al., 2011 [129]	Secondary analysis	South Africa	Rural, urban	2469	1–9	Male, female	Prevalence	24% of children among obese younger mothers		WHO IOTF
23	Symington et al., 2016 [130]	Secondary analysis	South Africa	Rural, urban	519	3–9	Male, female	Prevalence	12.0%		WHO IOTF
24	Tathiah et al., 2013 [131]	Secondary analysis	South Africa	Rural	963	7–14	Female	Prevalence	9%	3.8%	IOTF
25	Zeelie et al., 2010 [119]	Cross-sectional	South Africa	Rural, Urban	232	5–19	Male, female	Prevalence	4.1% of the boys and 9.9% of the girls had a BMI above the cut-off points		IOTF
26	Adegoke et al., 2009 [70]	Cross-sectional	Nigeria	Semi-urban	720	6–18	Male, female	Prevalence	2.8%	0.3%	IOTF
27	Adesina et al., 2012 [76]	Cross-sectional	Nigeria	Urban	960	10–19	Male, female	Prevalence	6.3%	1.8%	BMI
28	Akodu et al., 2012 [120]	Cross-sectional	Nigeria	Urban	160	2–15	Male, female	Prevalence	Did not report on this	Hemoglobin genotype SS subjects = 2.5%, and hemoglobin genotype AA controls =3.8%	WHO
29	Ene-Obong et al., 2012 [121]	Cross-sectional	Nigeria	Urban	1599	5–18	Male, female	Prevalence	11.4%	2.8%	IOTF
30	Fetuga et al., 2011 [74]	Cross-sectional	Nigeria	Semi-urban	1690	6–16	Male, female	Prevalence	3.0%	Did not report on this	CDC WHO
31	Fetuga et al., 2011 [75]	Cross-sectional	Nigeria	Semi-urban	1016	6–10	Male, female	Prevalence	Did not report on this	0.5%	WHO
32	Maruf et al., 2013 [79]	Cross-sectional	Nigeria	Urban	9014	2–18	Male, female	Prevalence	6.1%	0.8%	IOTF
33	Musa et al., 2012 [78]	Cross-sectional	Nigeria	Rural, urban	3240	9–16	Male, female	Prevalence	9.7%	1.8%	IOTF
34	Nwaiwu et al., 2015 [122]	Cross-sectional	Nigeria	Not specified	406	2–15	Male, female	Prevalence	15.4%	Did report this	IOTF
35	Oduwole et al., 2012 [123]	Cross-sectional	Nigeria	Urban	885	9–18	Male, female	Prevalence	13.8%	9.4%	CDC
36	Okagua et al., 2016 [81]	Cross-sectional	Nigeria	Urban	2282	10–19	Male, female	Prevalence	14.6% (Females) 11.4% (Males)	5.2% (Females) 3.8%(Males)	WHO
37	Omisore et al., 2015 [80]	Cross-sectional	Nigeria	Not specified	1000	10–19	Male, female	Prevalence	10.2% (Males) 5.3% (Females)	3.9% (Males) 2.0% (Females)	IOTF
38	Omuemu et al., 2010 [71]	Cross-sectional	Nigeria	Urban	300	10–19	Male, female	Prevalence	5.7%		CDC
39	Opara et al., 2010 [72]	Cross-sectional	Nigeria	Rural, urban	985	2.5–14	Male, female	Prevalence	11.1 and 0.2% respectively in private and public schools		WHO
40	Senbanjo et al., 2010 [73]	Cross-sectional	Nigeria	Urban	570	5–19	Male, female	Prevalence	1.9%		WHO
41	Senbanjo et al., 2011 [114]	Cross-sectional	Nigeria	Urban	570	5–19	Male, female	Trend	Did not report on this	5.0% general obesity	WHO
42	Senbanjo et al., 2012 [77]	Cross-sectional	Nigeria	Urban	423	10–19	Male, female	Prevalence	Did not report on this	24.5% central obesity	WHO
43	Uwaezuoke et al., 2016 [124]	Cross-sectional	Nigeria	Urban	2419	10–19	Male, female	Prevalence	Did not report this	Twelve of 41 obese males (29.3%) and 30 of 96 obese females (31.3%)	BMI
44	Mekonnen et al., 2018 [82]	Cross-sectional	Ethiopia	Rural, urban	634	6–12	Male, female	Prevalence	8.8%	3.1%	WHO
45	Moges et al., 2018 [83]	Cross-sectional	Ethiopia	Urban	1276	10–19	Male, female	Prevalence	17.0%		WHO
46	Sorrie et al., 2017 [84]	Cross-sectional	Ethiopia	Urban	504	3–5	Male, female	Prevalence	13.8%		WHO
47	Tadesse et al., 2017 [85]	Cross-sectional	Ethiopia	Urban	462	3–6	Male, female	Prevalence	6.9%		WHO
48	Teshome et al., 2013 [87]	Cross-sectional	Ethiopia	Urban	559	10–19	Male, female	Prevalence	12.9%	2.7%	WHO
49	Wakayo et al., 2016 [86]	Cross-sectional	Ethiopia	Rural, urban	174	11–18	Male, female	Prevalence	10.3%		WHO
50	Pangani et al., 2016 [24]	Cross-sectional	Tanzania	Urban	1781	8–13	Male, female	Prevalence	15.9%	6.7%	WHO
51	Mosha et al., 2010 [88]	Cross-sectional	Tanzania	Urban	428	6–12	Male, female	Prevalence	Did not report on this	5.6% in Dodoma compared to 6.3% in Kinondoni municipalities	BMI
52	Muhihi et al., 2013 [16]	Cross-sectional	Tanzania	Rural, urban	446	6–17	Male, female	Prevalence	Did not report on this	Overall, 5.2% (6.3% in girls and 3.8% in boys)	IOTF
53	Mushengezi et al., 2014 [125]	Cross-sectional	Tanzania	Urban	582	12–19	Male, female	Prevalence	22.2%		WHO
54	Mwaikambo et al., 2015 [89]	Cross-sectional	Tanzania	Urban	1722	7–14	Male, female	Prevalence	10.2%	4.5%	IOTF
55	Adamo et al., 2011 [62]	Cross-sectional	Kenya	Rural, urban	179	9–13	Male, female	Prevalence	6.8% of boys and 16.7% of girls in urban Kenya		BMI
56	Gewa, 2010 [137]	DHS	Kenya	Rural, urban	1495	3–5	Male, female	Prevalence	18.0%	4.0%	WHO
57	Kimani-Murage et al., 2015 [90]	Cross-sectional	Kenya	Urban	3335	Under 5	Male, female	Prevalence	8.8%		WHO
58	Wachira et al., 2018 [91]	Cross-sectional	Kenya	Urban	563	9–11	Male, female	Prevalence	20.8%		WHO
59	Choukem et al., 2017 [93]	Cross-sectional	Cameroon	Urban	1343	3–13	Male, female	Prevalence	12.5% (13.2% in girls and 11.8% in boys)		WHO
60	Navti et al., 2014 [94]	Cross-sectional	Cameroon	Rural, urban	557	5–12	Male, female	Prevalence	17.0 and 17.8% in girls and boys respectively		WHO
61	Tchoubi et al., 2015 [132]	Secondary analysis	Cameroon	Rural, urban	4518	< 5	Male, female	Prevalence	8.0%		WHO
62	Wamba et al., 2013 [92]	Cross-sectional	Cameroon	Urban	2689	8–15	Male, female	Prevalence	Ranged from 6.4 to 8.2% in boys and from 10.7 to 17.2% in girls	Ranged from 1.4 to 5.5% in boys and from 2.4 to 8.6% in girls	IOTF WHO CDC BMI database
63	Adom et al., 2019 [96]	Cross-sectional	Ghana	Urban	543	8–11	Male, female	Prevalence	16.4%		WHO
64	Kumah et al., 2015 [97]	Cross-sectional	Ghana	Urban	500	10–20	Male, female	Prevalence	12.2%	0.8%	IOTF
65	Mohammed et al., 2012 [95]	Cross-sectional	Ghana	Urban	270	5–15	Male, female	Prevalence	Did not report on this	10.9% (Girls = 15.0%, Boys = 7.2%)	WHO
66	Dos Santos et al., 2014 [98]	Cross-sectional	Mozambique	Urban, suburban	3374	8–15	Male, female	Prevalence, Trend	5.0% (Boys) 11.2% (Girls)	6.0% (Boys) 9.1% (Girls)	WHO
67	Dos Santos et al., 2015 [99]	Cross-sectional	Mozambique	Urban, suburban	323	10–15	Male, female	Prevalence	7.5% (Boys) 21.0% (Girls)		IOTF
68	Nagwa et al., 2011 [100]	Cross-sectional	Sudan	Urban	1138	10–18	Male, female	Prevalence	10.8%	9.7%	WHO
69	Salman et al., 2011 [101]	Cross-sectional	Sudan	Urban	304	6–12	Male, female	Prevalence	14.8%	10.5%	CDC
70	Christoph et al., 2017 [102]	Cross-sectional	Uganda	Rural, urban	148	11–16	Male, female	Prevalence	1.4%		WHO
71	Turi et al., 2013 [133]	Secondary analysis	Uganda	Rural, urban	1099	< 5	Male, female	Prevalence	13.5%		WHO
72	Wrotniak et al., 2012 [103]	Cross-sectional	Botswana	Rural, urban	707	12–18	Male, female	Prevalence	12.3%	5.0%	WHO
73	Juwara et al., 2016 [104]	Cross-sectional	Gambia	Urban	960	13–15	Male, female	Prevalence	22.8% in private schools and 4.5% in public schools		WHO
74	Van den Berg et al., 2014 [105]	Cross-sectional	Lesotho	Urban	221	16	Male, female	Prevalence	8.3% of boys and 27.2% of girls		WHO CDC IOTF
75	Caleyachetty et al., 2012 [106]	Cross-sectional	Mauritius	Rural, urban	241	9–10	Male, female	Prevalence	15.8% in boys and 18.9% in girls	4.9% in boys and 5.1% in girls	IOTF
76	Bovet et al., 2010 [107]	Cross-sectional	Seychelles	Rural, urban	8462	Mean ages; 9.2, 12.6 and 15.3 years	Male, female	Prevalence	37% of boys in private schools compared to 15% in public schools 33% of girls in private compared to 20% of those in public schools		IOTF
77	Sagbo et al., 2018 [108]	Cross-sectional	Togo	Urban	634	8–17	Male, female	Prevalence	5.2%	1.9%	IOTF
78	Kambondo et al., 2018 [109]	Cross-sectional	Zimbabwe	Rural, urban	974	6–12	Male, female	Prevalence	Did not report on this	13.8% in urban compared to 2.3% in rural areas	IOTF
79	Muthuri et al., 2016 [110]	Cross-sectional	Kenya, South Africa	Rural, urban	4725	9–11	Male, female	Prevalence	18.8 and 30.6% in Kenya and South Africa respectively	Did not report on this	WHO
80	Peltzer et al., 2011 [128]	Secondary analysis	Ghana, Uganda	Not specified	5613	13–15	Male, female	Prevalence	10.4% (Girls) and 3.2% (Boys)	0.9% (Girls) and 0.5% (Boys)	IOTF
81	Manyanga et al., 2014 [111]	Cross-sectional	Benin, Ghana, Mauritania and Malawi	Not specified	23,496	11–17	Male, female	Prevalence	8.7% in Ghana, 10.0% in Malawi, 11.2% in Benin, and 24.3% in Mauritania		WHO

### Study findings

In all, 80 of the 81 included studies reported findings on the prevalence of childhood overweight/obesity, 8 on trends of childhood overweight/obesity, and one reported on the incidence of childhood overweight/obesity.

### Prevalence of childhood overweight/obesity

Most (*n* = 80) of the included studies reported evidence on the prevalence of obesity/overweight among children and adolescents. Armstrong et.al. in South Africa involving 24,391 school children aged from 8 to 11 years, reported 13 and 3.3% childhood prevalence of overweight and obesity respectively in 2004 [63]. Armstrong et.al. noted that these findings were much higher than the baseline values of 1.2 and 0.2% prevalence of childhood overweight and obesity respectively in 1994 [63]. Again, Armstrong et.al [64]. study involving school children aged 6 to 13 years old reported a combined overweight and obesity prevalence of 15.4% (1564/10,285). Craig et al. [65] study compared different international standards of assessing body composition in rural KwaZulu-Natal. Using WHO 2007 reference criteria, the combined overweight/obesity prevalence was 9.2, 8.1, and 8.0% in males compared to 13.6, 13.4, and 25.8% in females aged 7, 11, and 15 years respectively [65]. These findings however differed substantially when Cole et al. and IOFT cut-offs were used, with reported combined childhood obesity and overweight prevalence of 3.2, 5.2, and 6.1% in males compared to 9.2, 9.7, and 22.7% in females within the respective age (7, 11, 15 years) distributions [65]. Kimani-Murage et al. in rural Mpumalanga Province, found a prevalence of overweight/obesity of 4% in boys and 15% in girls among 1848 adolescents aged 10 to 20 years [66]. Ngwenya et al. studied 175 adolescents from the urban city of Tshwane and identified 15.4% (*n* = 27) overweight and 8.6% (*n* = 15) obesity prevalence [67]. In the Western Cape, Negash et al. reported an overall prevalence of overweight/obesity of 22.9% with girls having significantly higher levels (19.7%/9.1%) than boys (9.4%/4.5%) and Whites learners being more overweight (20.7%) while Coloured learners were more obese (7.5%) [68]. Similarly, Feeley et al. recorded a combined overweight/obesity prevalence of 8.1 and 27.0% in males and females respectively, while Ginsburg et al. reported a combined overweight/obesity prevalence of 8.0 and 25.0% for males and females respectively, albeit with different sample sizes [134, 135]. Pretorius et al. and Symington et al. reported 27.3 and 12.0% prevalence of overweight/obesity in their respective studies of rural-urban populations in South Africa [69, 130]. Using WHO growth standards, Lesiapeto et al. identified a combined overweight/obesity prevalence of 16.1% among 2485 children under 5 years in two rural districts of South Africa [126]. Additionally, a case study conducted by Mokabane et al. among 56 black girls aged 13 to 19 years in a peri-urban area of Limpopo Province identified a 12.5% overweight and 3.6% obesity prevalence [139].

Of the 18 studies conducted in Nigeria, 17 provided evidence on childhood overweight/obesity prevalence [70–84, 86, 87]. A study by Adegoke et al. in 2009 aimed to determine the nutritional status of children aged from 6 to18 years in Ile-Ife observed a prevalence of 0.3% (2/720) obesity (both being female), and 2.8% (20/720) overweight (of which 15 (85%) were female) [70]. Omuemu et al. study in an urban city of Edo State reported 5.7% overweight/obesity [71]; Opara et al. reported 11.1 and 0.2% of overweight/obesity respectively in private and public schools in Uyo [72], and Senbanjo et al. reported 1.9% prevalence of overweight/obesity in Abeokuta [73]. Fetuga et al. cross-sectional surveys involving school children aged 6 to10 years in the semi-urban town of Sagamu, Ogun State reported 3.0% overweight and 0.5% obesity using the WHO reference [74, 75]. In their respective urban studies in 2012 among adolescents 10 to 19 years, Adesina et al. found overweight and obesity prevalence of 6.3 and 1.8% in Port Harcourt; while in Abeokuta, Senbanjo et al. observed a 5% prevalence of general obesity and 24.5% central obesity [76, 77]. Similarly, Musa et al. reported overweight and obesity prevalence of 9.7 and 1.8% respectively among adolescents in Benue State [78]. Maruf et al. reported an overall prevalence of 6.1% overweight and 0.8% obesity respectively in their cross-sectional study of Nigerian school children and adolescents aged 2 to 18 years [79]. Omisore et al. observed a significantly higher proportion of overweight and obesity in females (10.2 and 3.9%) than males (5.3 and 2.0%) in their 2015 study of 1000 adolescents aged 10 to 19 years in Osun State [80]. Okagua et al. study in Port Harcourt also reported a higher prevalence of overweight and obesity in females (14.6 and 5.2%) than males (11.4 and 3.8%) among adolescents aged 10 to 19 years [81].

In Ethiopia, all six included studies were cross-sectional surveys that reported evidence on childhood obesity/overweight prevalence. Mekonnen et al. study in Bahir Dar City among 634 school children aged 6 to 12 years reported an overall overweight/obesity prevalence of 11.9% (8.8% overweight and 3.1% obesity) [82]; while Moges et al. reported the prevalence of overweight/obesity to be 17.0% among 1276 adolescents aged 10 to 19 years in Addis Ababa [83]. Sorrie et al. and Tadesse et al. identified a combined overweight/obesity prevalence of 13.8% in Gondar City and 6.9% in Bahir Dar City respectively in their studies involving pre-school children [84, 85]. Wakayo et al. study in central Ethiopia reported an overweight and/or obesity prevalence of 10.3% among schooling adolescents aged 11 to 18 years old [86]. Teshome et al. also found 12.9% overweight and 2.7% obesity prevalence in their study involving 559 high school adolescents aged 10 to 19 years in the urban city of Hawassa [87].

In Tanzania, a comparative cross-sectional study by Mosha et al. in 2010 found that the prevalence of obesity among children 6 to 9 years was 5.6% in Dodoma compared to 6.3% in Kinondoni municipalities; and for those aged 10 to 12 years, the prevalence of obesity was 3.9% compared to 5.8% in Dodoma and Kinondoni municipalities respectively [88]. Muhuhi et al. study in Dar es Salaam [16] reported a 5.2% overall prevalence of obesity (6.3% in girls and 3.8% in boys). Mwaikambo et al. also in Dar es Salaam reported 10.2% overweight and 4.5% obesity rates among 1722 children aged 7 to 14 years [89]. Pangani et al. also studied 1781 primary school children aged 8–13 years and identified prevalence of overweight and obesity to be 15.9 and 6.7% respectively [24].

Four studies each from Kenya and Cameroon reported evidence of childhood obesity and/or overweight prevalence. Using data collected in the 2003 nationwide DHS in Kenya, Gewa et al. in 2009 reported approximately 18.0% overweight and 4.0% obesity prevalence from a random sample of 1495 pre-school children aged 3 to 5 years [137]. Adamo et al. study compared rural and urban Kenyan children (*n* = 179, 9–13 years) and observed that whereas none of the rural Kenyan children were neither overweight nor obese, 6.8% of boys and 16.7% of girls in urban Kenya were found to be either overweight or obese [62]. In an urban poor setting of Nairobi, Kimani-Murage et al. [90] reported 8.8% overweight/obesity prevalence among a cohort of 3335 children under five. Wachira et al. examined 563 children aged 9 to 11 years attending 29 non-boarding primary schools in Nairobi, as part of the International Study of Childhood Obesity, Lifestyle and the Environment (ISCOLE) and found that 20.8% of participants were either overweight or obese [91]. In the urban city of Douala, Cameroon, Wamba and colleagues employed different international references and reported significant differences among the methods with a prevalence of overweight ranging from 6.4 to 8.2% in boys and from 10.7 to 17.2% in girls; whereas the prevalence of obesity ranged from 1.4 to 5.5% in boys and from 2.4 to 8.6% in girls [92]. A similar study in Douala by Choukem et al. utilized WHO BMI-for-age reference curves and observed that prevalence of obesity/overweight was 12.5% (13.2% in girls and 11.8% in boys) [93]. Navti and colleagues studied 557 school children aged 5 to 12 years from both rural and urban areas of northwest Cameroon and found the prevalence of overweight/obesity to be 17.0 and 17.8% in girls and boys respectively [94]. The prevalence of obesity/overweight was 8.0% among 4518 children under 5 years in rural and urban Cameroon as reported by Tchoubi et al. [132].

Studies from three urban areas in Ghana were included in this study. In the city of Accra, Mohammed and Vuvor reported obesity prevalence of 10.9% (girls = 15.0%, boys = 7.2%) among 270 basic school children between 5 and 15 years [95]; while Adom and colleagues reported an overall overweight/obesity prevalence of 16.4% among school children aged 8 to11 years in Adentan municipality in 2019 [96]. Kumah et al. in 2015 also reported an overweight and obesity prevalence of 12.2 and 0.8% respectively from a sample of 500 high school students in Kumasi [97].

Two studies each were conducted in Mozambique, Sudan, and Uganda. A study by Dos Santos and colleagues in Maputo reported the prevalence of overweight/obesity to be 5.0%/6.0% in boys and 11.2%/9.1% in girls [98]. Dos Santos et al. study also in Maputo among 323 adolescents aged 10 to 15 years reported the prevalence of overweight/obesity to be 7.5% in boys and 21.0% in girls [99]. In the Khartoum State of Sudan, Nagwa et al. observed that, the prevalence of overweight and obesity were 10.8 and 9.7% in adolescents 10 to 18 years old [100]; while Salman et al. found 14.8% overweight and 10.5% obesity among children between 6 and 12 years [101]. In Uganda, Turi et al. analyzed data from the 2011 DHS and found that 13.5% of children under 5 years were either overweight or obese [133]. Christoph et al. piloted a survey measuring weight-related factors in 148 (11–16 years) rural and urban school children in Uganda and recorded an overweight and obesity prevalence rate of 1.4% [102].

Of the 81 studies included in this review, seven countries, Botswana, Gambia, Lesotho, Mauritius, Seychelles, Togo, and Zimbabwe reported one study each providing evidence on childhood overweight/obesity. In a cross-sectional study, Wrotniak et al. identified overweight and obesity prevalence of 12.3 and 5.0% respectively in Botswana [103]. Juwara et al. recorded an overall overweight/obesity prevalence of 22.8% in private schools and 4.5% in public schools among adolescents in urban Gambia [104]. Among 225 16-year olds in urban Maseru, Lesotho, van den Berg et al. reported that 8.3% of boys and 27.2% of girls were overweight and/or obese [105]. In Mauritius, Caleyachetty et al. found the prevalence of overweight to be 15.8% in boys and 18.9% in girls while obesity was 4.9% in boys and 5.1% in girls [106]. Bovet et al. study in Seychelles reported that 37% of boys in private schools compared to 15% in public schools were overweight/obesity, whereas 33% of girls in private compared to 20% of those in public schools were either overweight or obese [107]. Sagbo et al. observed 5.2 and 1.9% prevalence of overweight and obesity respectively in urban areas of Lomé [108]. In Zimbabwe, Kambondo and colleagues studied 974 children aged 6 to 12 years and reported an obesity prevalence of 13.8% in urban compared to 2.3% in rural areas [109].

Three out of the 81 included studies were from multi-country settings. Muthuri et al. utilized data from a 12-country study (ISCOLE) which included two SSA countries and reported an overweight prevalence of 18.8 and 30.6% in Kenya and South Africa respectively [110]. Peltzer and colleague performed a secondary analysis of existing data from the Global School-Based Health Survey (GSHS) from two SSA countries (Ghana and Uganda) in which they recorded prevalence of overweight/obesity of 10.4% in girls and 3.2% in boys, with 0.9 and 0.5% obesity among girls and boys, respectively [128]. Moreover, Mayanga et al. also analyzed data from 7 countries involved in the GSHS, out of which 4 were in SSA and reported a combined overweight/obesity prevalence 8.7% in Ghana, 10.0% in Malawi, 11.2% in Benin, and 24.3% in Mauritania [111].

### Incidence of childhood overweight/obesity

Of the 81 included studies only one study presented evidence on the incidence of childhood overweight and obesity. Lundeen et al. study in South Africa aimed to describe the gender differences in overweight and obesity from infancy to late adolescence among a cohort showed that the incidence of obesity was highest from 4 to 8 years to11–12 years in boys (6.8 cases per 1000 person-years) and from 11 to 12 years to 13–15 years in girls (11.2 cases per 1000 person-years) [127]. This finding suggests limited studies focusing on the incidence of childhood obesity in SSA.

### Trends of childhood overweight/obesity

Eight of the included studies reported evidence of childhood obesity and overweight trends in SSA. Of these, 6 (75%) were conducted in South Africa, and one each (12.5%) in Nigeria and Mozambique. In South Africa, Armstrong et al. compared data across two time periods (1994 and 2001/2004) and observed increasing secular trends of overweight and obesity from 1.2 to 13.0% and 0.2 to 3.3% among young South Africans over the 7–10 years period [63]. Feeley et al. collected anthropometric data from a birth cohort of 1298 children in the Soweto at age 13, 15, and 17 years and found that the combined overweight and obesity at 17 years was respectively 8.1 and 27% among males and females indicating a decrease in males and an increase in females from when they were 13 years old, though it was not stated by what magnitudes [134]. Kruger et al. study reported that the combined prevalence of overweight/obesity based on BMI cut-offs decreased significantly from 17.1% in 1999 to 14.0% in 2005 [112]. Lundeen and colleagues reported that obesity and overweight prevalence declined from age 1–2 years to 16–18 years among boys whereas among girls overweight and obesity prevalence increased throughout childhood (from 4 to 6 years to 16–18 years) among a cohort of 1172 children [127]. Pienaar et al. investigated changes in overweight and obesity prevalence among 574 children aged 6 to 9 years and found that obesity increased over the 3-years by 4% from 12.5% at baseline to 16.7% during follow-up [15]. This change was higher in whites (4.2%) than blacks (2.0%) and in boys (3.2%) compared to girls (2.4%). Reddy et al. [113] also observed that overweight rates increased from 6.3% in 2002 to 11.0% in 2008 among male adolescents and from 24.3% in 2002 to 29.0% in 2008 among female adolescents; while obesity rates more than doubled among male adolescents from 1.6% in 2002 to 3.3% in 2008 compared to a rise from 5.0 to 7.5% among females who participated in the South African National Youth Risk Behaviour Survey in 2002 and 2008. Senbanjo et al. study aimed to determine current nutritional status and its changes between 1983 and 2006 among school children and adolescents in Abeokuta, Nigeria found that obesity prevalence rose from 1.7 to 3.3% in males and from 2.6 to 5.1% in females over the period [114]. In Mozambique, Dos Santos et al. examined secular trends in the nutritional status of children and adolescents for 1992, 1999, and 2012 and reported that obesity increased from 0.8 to 1.6 and 6.0 respectively in boys and from 1.8 to 4.5 and 9.1 respectively in girls [98]. This finding also suggests limited studies focusing on trends of childhood overweight obesity in SSA.

## Discussion

This scoping review presented evidence on the prevalence, incidence, and trends of childhood obesity in SSA from studies published between January 2009 and June 2019. The review showed that a total of 81 studies were published within the period from 17 SSA countries including 3 multi-country studies. Most (53%) of the studies were conducted in South Africa (31%) and Nigeria (22%) with 11 countries reporting less than 5 studies each. Moreover, the majority (81.5%) of the studies were cross-sectional and most studies (79) focused on both male and female participants with no study reporting on only male participants. The review further revealed that almost all the included studies (80/81) reported about prevalence with only 1 study reporting about incidence while 8 reported on trends. This study’s findings suggest limited evidence on trends and incidence of childhood obesity in SSA.

We found 81 studies presenting evidence on the burden of childhood obesity published in 20 SSA countries between 2009 and July 2019. This points to a lack of evidence from over 60% of the countries included in the WHO list of SSA countries [140, 141], which presents huge literature gaps. This supports findings from the systematic review by Keino et al. which explored the determinants of stunting and overweight in SSA and reported a paucity of literature from most parts of SSA [49]. It further backs findings from Jaacks et al. systematic review of current evidence on maternal and child overweight and obesity in the context of undernutrition which indicated limited scientific literature, especially in LMIC to support such reviews [54].

The highest number of studies were reported in South Africa (31%) which shows a high level of interest in childhood obesity research in that country. This interest may stem from the fact that several studies have reported that South Africa leads on the league table of countries with the highest prevalence of obesity in Africa [27, 142]. The WHO Global Status Report on NCDs, 2010 indicates that the prevalence of overweight is highest in upper-middle-income countries (UMICs) while the fastest rise in overweight is in lower-middle-income countries [143]. Agreeably, most of the included studies in this review, 74.1% (60/81) were from either UMIC whilst 21 and 1.2% were from LMIC and low-income countries respectively with only one study (1.2%) was from a high-income country (Seychelles) based on the 2019–2020 World Bank country classifications [144] (Table 3).

**Table 3:** 2019–2020 World Bank country income level classifications of the countries the included studies were conducted (*N* = 20)

High-income country	Upper-middle-income countries	Lower-middle-income countries	Low-income countries
Seychelles	Botswana	Cameroon	Ethiopia
	Mauritius	Ghana	Mozambique
	South Africa	Kenya	Tanzania
		Lesotho	Togo
		Nigeria	Uganda
		Sudan	Malawi
		Zimbabwe	Benin
		Mauritania	The Gambia
**Total = 1**	**Total = 3**	**Total = 8**	**Total = 8**

We further observed that majority of the included studies utilized either WHO growth references (48.2%) or Cole et al. and IOTF criteria (34.6%) with few employing BMI cut-off (7.6%) and CDC classifications (3.7%). This agrees with findings from systematic reviews by Monyeki et al. and Muthuri et al. which stated that most of the studies in their review employed different widely accepted (Cole et al. and IOTF, CDC and WHO) international cut-off points [20, 51]. However, Monyeki et al. further stated that the Cole-IOTF and WHO 2006 growth standards tend to overestimate overweight and obesity prevalence hence making it difficult to interpret findings across studies due to the variations in these reference standards [51]. In contrast, Bentham et al. study aimed to determine worldwide trends in mean BMI in children and adolescents reported that prevalence using WHO criteria were higher than those of IOTF and CDC but yielded similar trends [5], and this tends to agree with findings from this study. We, therefore, recommend that future researches aim at comparing childhood overweight and obesity prevalence should be based on a single reference criterion to ensure uniformity and comparability of data.

Among children under 5 years, the combined prevalence of overweight and obesity reported ranged from a minimum of 8.0% in Cameroon [132] to a maximum of 16.0% in South Africa [126]. This confirms reports by UNICEF, WHO, and the World Bank which ranked Southern Africa as the region with the highest prevalence of overweight among children under 5 years (14.6%), followed by Central Asia (11.6%) and Northern Africa (11.0%) [145]. This finding presents serious policy implications and the urgent need to implement interventions to reverse this trend and forestall future adverse consequences. Moreover, the highest reported prevalences among children and adolescents were in Seychelles, South Africa, Lesotho, and Mauritania. In contrast, Uganda, Cameroon, Tanzania, and Nigeria recorded some of the lowest prevalence. There was a higher prevalence of overweight/obesity among girls than boys in private than in public schools, and in urban than in rural areas in most of the study settings. This contrasts with findings by Duncan et al. in which the prevalence of overweight and obesity was higher among males than females in Brazil [146]. Similarly, Adamo et al. who compared children from rural and urban Kenya to their counterparts in Canada reported that while obesity/overweight was non-existent in the rural Kenyan population, urban Kenyan children were anthropometrically similar to their contemporaries in Canada [62]. This suggests a possible nutritional transitional in urban areas which may be partly explained by the adoption of more western and obesogenic lifestyles by urban dwellers in SSA. These findings again point to an obvious fact that obesity and overweight rates in the region are currently comparable and in some instances exceed rates in highly developed countries; where reported prevalence range from 10% in Denmark to 31% in the United State [147].

Our findings also revealed rapidly increasing trends of childhood overweight and obesity in SSA particularly among adolescent girls [111, 130, 136]. This supports reports by Tzioumis and colleagues that the global prevalence of obesity has increased in all regions of the world with developing countries recording greater absolute numbers of affected children and higher relative increases [148]. According to the WHO, while most countries in the world are experiencing a rapid upsurge in childhood obesity, this situation is even more alarming in LMICs, especially in SSA where the “dual burden” of infectious diseases and under-nutrition co-existing with high rates of NCDs risk factors such as obesity and overweight is prevalent [6, 140, 141]. Ng et al. further reported after their systematic analysis of the Global Burden of Disease study from 1980 to 2013 that, not only is obesity increasing but no national success stories have been reported in the past 33 years [142]. Lobstein and Jackson-Leach in 2016 estimated that by 2025 some 268 million children aged 5–17 years globally may be overweight, including 91 million obese based on the assumption that no policy interventions prove effective at changing current trends [21]. They hence concluded that the WHOs Sustainable Development Goal (SDG) target to halt the rise in obesity by 2025 and reduce premature mortality due to NCDs by one third by the year 2030 is unlikely to be met and that health service providers will need to plan for a significant increase in obesity-related comorbidities. Bollyky et al. have indicated elsewhere that lower-income countries that face the most rapid surge in NCD burden are also the least prepared and are expected to make the least increases in health expenditure [143].

### Implications for research

We observed a paucity of literature on the outcomes, especially on incidence and trends of childhood obesity and overweight from most countries in the SSA region. The studies also utilized different anthropometric methods of assessment which made it difficult for comparisons. Moreover, the studies which reported on trends were not very current. We, therefore, recommend that (i) more studies should be conducted especially on time trends and incidence of childhood obesity/overweight in different setting across SSA, (ii) future reviews should focus on studies utilizing one specific assessment criterion for easy interpretation and comparability of results, (iii) the finding of the study presents alarming high prevalence and rising trends of childhood obesity and overweight hence, we recommend more intervention studies that will develop practicable solutions to address this challenge, and (iv) we also recommend follow-up studies to map evidence on the risk factors of childhood obesity in SSA, in order to guide efforts aimed at preventing the problem.

### Implications for practice

This study findings demonstrate an urgent need for a more concerted effort among governments in SSA and the global health community in tackling the rising burden of obesity/overweight and NCDs in the region. To this end, policymakers are encouraged to formulate policies that facilitate the identification, management, and most especially prevention of childhood overweight/obesity. Governments in SSA should also endeavor to increase funds and resource allocation towards combating this problem which will potentially reduce the NCD burden in the region. Finally, policy interventions should be holistic, context-specific, age-appropriate, culturally and socially responsive, and multi-sectorial in nature to increase their chances of success.

### Strengths and limitations of the study

We followed all the steps required of systematic reviews in reporting this study except the registration in PROSPERO. The study protocol was however published in a peer-reviewed journal [31]. We also performed a thorough, systematic, and comprehensive search for literature on the prevalence, incidence, and trends of childhood obesity in SSA using MeSH terms to address alternative terminologies of the keywords. This study also had many limitations. This study included only published articles. This potentially excluded relevant information that may have been documented on national and international organizations registries or websites such as the WHO, ministries of health, government statistical service, and other grey literature. The language, and geographical setting limitations perhaps, exclude important evidence hence affecting the external generalization of the results. For example, four studies that were published in French but provided abstracts in English were able to pass the abstract screening stage but were dropped at full-text screening stage due to lack of expertise to translate the French language. This study also sought to provide recent evidence (10 years) hence, the date limitation from 2009 to 2019 probably excluded useful sources of evidence. As part of this study’s published protocol [31], we planned to appraisal the methodological quality of the included studies, but this was not done due to the explorative nature of scoping reviews. Unlike systematic reviews and meta-analysis, quality appraisal is also not mandatory for a scoping review study. Moreover, the risk of bias might not be useful considering the number of included studies. Nonetheless, we hope to conduct a full systematic review and meta-analysis as a follow-up study. Hence, we will perform the methodological quality appraisal and additionally report the risk of bias in the next phase of this study.

## Conclusion

Our study findings indicate increasing prevalence, incidence, and trends of childhood obesity in SSA. However, this study suggests limited studies focusing on childhood obesity/overweight in most SSA countries. The evidence demonstrated by this review should, therefore, serve as a wake-up call for researchers in SSA and the global health community to expedite action through proactive and pragmatic interventions to stem the rise of childhood obesity/overweight and consequent NCD burden in SSA. Finally, we reiterate the statement by the Pan American Health Organization that although the cost of NCDs is high, the cost of inaction is even higher and that paying for NCD prevention and control is much more than a cost, but rather, an investment for the future [140].

## Supplementary Information

**Additional file 1.** The Preferred Reporting Items for Systematic reviews and Meta-Analyses extension for Scoping Reviews (PRISMA-ScR) Checklist.

**Additional file 2.** Electronic databases search results for title screening.

## Data Availability

The data supporting the conclusion of this paper are available through the detailed reference list. No original datasets are presented since this was a review of previously existing literature.

## Abbreviations

BMI: Body Mass Index
CDC: Centers for Disease Control and Prevention
DHS: Demographic and Health Survey
GBD: Global Burden of Disease
GSHS: Global School-Based Health Survey
IOTF: International Obesity Task Force
ISCOLE: International Study of Childhood Obesity, Lifestyle and Environment
LIC: Low-Income Country
LMIC: Lower-Middle-Income Country
MMAT: Mixed Methods Appraisal Tool
NCD: Non-Communicable Disease
NCHS: National Center for Health Statistics
PEO: Population, Exposure and Outcomes
PRISMA-ScR: Preferred Reporting Items for Systematic Reviews and Meta-Analyses
SDG: Sustainable Development Goal
SSA: Sub-Saharan Africa
UMIC: Upper-Middle-Income Country
WHO: World Health Organization
