# Supplementary material for: Prevalence, incidence, and trends of childhood overweight/obesity in Sub-Saharan Africa: a systematic scoping review
Source: Arch Public Health. 2020 Oct 29;78:109. doi: 10.1186/s13690-020-00491-2 (PMC7599109; doi:10.1186/s13690-020-00491-2)
Supplement: Supplementary file 2 — Additional file 2. Electronic databases search results for title screening. [file 13690_2020_491_MOESM2_ESM.docx]

## Supplementary file 1: Electronic databases search results for title screening

| **Date** | **Databases** | **Keywords** | **Search results** | **Number of eligible titles** |
| --- | --- | --- | --- | --- |
| 19/05/2019 | PubMed | "obesity"[MeSH Terms] OR "obesity"[All Fields] OR "obese"[All Fields] OR ("overweight"[MeSH Terms] OR "overweight"[All Fields] OR "body mass index"[MeSH Terms] OR "body"[All Fields] OR "mass"[All Fields] OR "index"[All Fields] OR "body mass index"[All Fields]) OR bmi[All Fields] AND "child"[MeSH Terms] OR "child"[All Fields] OR "children"[All Fields] OR "childhood"[All Fields] OR "adolescent"[MeSH Terms] OR "adolescent"[All Fields] OR "adolescents"[All Fields] OR "adolescence"[All Fields] OR "youth"[All Fields] OR "pediatrics"[MeSH Terms] OR "pediatrics"[All Fields] OR "pediatric"[All Fields] OR "paediatric"[All Fields] OR "paediatrics"[All Fields] AND ("africa south of the sahara"[MeSH Terms] OR "africa south of the sahara"[All Fields] OR "sub Saharan Africa"[All Fields] OR "sub-Saharan Africa"[All Fields] OR "africa"[MeSH Terms] OR "africa"[All Fields] OR "Angola"[All Fields] OR "Benin"[All Fields] OR "Botswana"[All Fields] OR "Burkina Faso"[All Fields] OR "Burundi"[All Fields] OR "Cameroon"[All Fields] OR "Cape Verde"[All Fields] OR "Central African Republic"[All Fields] OR "Chad"[All Fields] OR "Comoros"[All Fields] OR "Congo"[All Fields] OR "Cote d'Ivoire"[All Fields] OR "Djibouti"[All Fields] OR "Equatorial Guinea"[All Fields] OR "Eritrea"[All Fields] OR "Ethiopia"[All Fields] OR "Gabon"[All Fields] OR "The Gambia"[All Fields] OR "Ghana"[All Fields] OR "Guinea"[All Fields] OR "Guinea-Bissau"[All Fields] OR "Kenya"[All Fields] OR "Lesotho"[All Fields] OR "Liberia"[All Fields] OR "Madagascar"[All Fields] OR "Malawi"[All Fields] OR "Mali"[All Fields] OR "Mauritania"[All Fields] OR "Mauritius"[All Fields] OR "Mozambique"[All Fields] OR "Namibia"[All Fields] OR "Niger"[All Fields] OR "Nigeria"[All Fields] OR "Reunion"[All Fields] OR "Rwanda"[All Fields] OR "Sao Tome and Principe"[All Fields] OR "Senegal"[All Fields] OR "Seychelles"[All Fields] OR "Sierra Leone"[All Fields] OR "Somalia"[All Fields] OR "South Africa"[All Fields] OR "Sudan"[All Fields] OR "Swaziland"[All Fields] OR "Tanzania"[All Fields] OR "Togo"[All Fields] OR "Uganda"[All Fields] OR "Western Sahara"[All Fields] OR "Zambia"[All Fields] OR "Zimbabwe"[All Fields] AND "humans"[MeSH Terms]) AND (("2009/01/01"[PDAT] : "2019/06/31"[PDAT]) AND "humans"[MeSH Terms]) | 60,021 | 447 |
| 21/05/2019 | Google Scholar | "obesity" OR "obese" OR "overweight" OR "body mass index" OR "body mass" OR bmi AND "child" OR "children"OR childhood" OR "adolescents" OR "adolescence" OR "youth" OR "pediatrics" OR "paediatric" OR "paediatrics" AND "africa" | 79,300 | 374 |
| 03/06/2019 | Web of Science | "obesity" OR "obese" OR "overweight" OR "body mass index" OR "body mass" OR bmi AND "child" OR "children"OR childhood" OR "adolescents" OR "adolescence" OR "youth" OR "pediatrics" OR "paediatric" OR "paediatrics" AND "africa" | 230 | 25 |
| 05/06/2019 | CINAHL via EBSCOhost | "obesity" OR "obese" OR "overweight" OR "body mass index" OR "body mass" OR bmi AND "child" OR "children"OR childhood" OR "adolescents" OR "adolescence" OR "youth" OR "pediatrics" OR "paediatric" OR "paediatrics" AND "africa" | 110,597 | 113 |
| **Total** |  |  | **250,148** | **959** |
